# Dexmedetomidine Infusion Associated with Transient Adrenal Insufficiency in a Pediatric Patient: A Case Report

**DOI:** 10.1155/2013/207907

**Published:** 2013-05-16

**Authors:** Elizabeth W. Tucker, David W. Cooke, Sapna R. Kudchadkar, Sybil Ann Klaus

**Affiliations:** ^1^Department of Anesthesiology and Critical Care Medicine, Pediatric Critical Care Medicine Fellowship, Johns Hopkins University School of Medicine, Charlotte Bloomberg Children's Center, 1800 Orleans Street, Baltimore, MD 21287, USA; ^2^Department of Pediatrics, Division of Pediatric Endocrinology, Johns Hopkins University School of Medicine, Baltimore, MD 21287, USA; ^3^Department of Anesthesiology and Critical Care Medicine, Division of Pediatric Anesthesiology and Critical Care Medicine, Johns Hopkins University School of Medicine, Baltimore, MD 21287, USA; ^4^Department of Pediatrics, Johns Hopkins University School of Medicine, Baltimore, MD 21287, USA

## Abstract

Dexmedetomidine is a highly selective **α**_2_-adrenoceptor agonist used for sedation due to its anxiolytic and analgesic properties without respiratory compromise. Due to its structural similarity to etomidate, there has been concern that dexmedetomidine may cause adrenal insufficiency. This concern was initially supported by animal studies, but subsequent human studies demonstrated mixed results. We describe the case of transient adrenal insufficiency in a 1-year-old male who presented with 24% total body surface 2nd degree burns. He required sedation with a prolonged, high-dose dexmedetomidine infusion with a peak infusion dose of 2.7 mcg/kg/hr and duration of 6.5 days. The patient developed lethargy and hypotension four days after discontinuation of his infusion. He had a random cortisol level which was low at 0.4 mcg/dL, and the concern for adrenal suppression was confirmed with an ACTH stimulation test with the baseline cortisol of 0.4 mcg/dL and inappropriate 60 minute post-ACTH stimulation cortisol of 7.8 mcg/dL. While further studies will be needed to clarify the risk of adrenal suppression secondary to dexmedetomidine, this case suggests that caution should be taken when administering dexmedetomidine to pediatric patients and highlights the need for future studies to look at appropriate dosing and duration of dexmedetomidine infusions.

## 1. Introduction

Dexmedetomidine is a highly selective *α*_2_-adrenoceptor agonist whose use in pediatrics has increased since its approval by the Food and Drug Administration (FDA) for sedation in adults [1]. Unlike other sedative medications that act on the *γ*-amino butyric acid (GABA) receptor, it works to provide sedation resembling natural sleep, anxiolysis, analgesia, reduced delirium, sympatholysis, and antishivering properties without respiratory depression [2–7]. The presumed safety of dexmedetomidine has translated to its use in several settings: in pediatric and adult intensive care units for sedation and sedative weaning, in operating rooms to attenuate the cardiovascular and neuroendocrine responses to surgery by decreasing sympathetic activity, and in outpatient radiologic centers for imaging sedation with low risk of respiratory compromise [1, 2, 4–7]. 

Because of dexmedetomidine's structural similarity to etomidate, there has been concern that dexmedetomidine could cause adrenal insufficiency [8]. Etomidate is known to cause adrenal insufficiency by inhibiting 11*β*-hydroxylase and side chain cleavage enzymes, thereby inhibiting adrenal steroidogenesis [9]. The mechanism of this enzyme inhibition is dependent on the imidazole ring structure of etomidate, a structure also contained within dexmedetomidine [8]. Indeed, initial in vivo and in vitro animal studies demonstrated decreased cortisol levels and blunted cortisol response to adrenocorticotrophic hormone (ACTH) stimulation after dexmedetomidine administration [8]. However, human studies have demonstrated mixed results: an adult study comparing dexmedetomidine and propofol showed no difference in cortisol and ACTH concentrations between treatment groups, but another adult study in gynecologic laparoscopy demonstrated that intramuscular dexmedetomidine decreased cortisol levels in a dose-dependent manner [10, 11]. To our knowledge, there have been no studies to date investigating the effect of dexmedetomidine on adrenal steroidogenesis in the pediatric population. We report a case of transient adrenal insufficiency in a pediatric patient after prolonged, high-dose dexmedetomidine infusion was used to wean opioid and benzodiazepines and facilitate extubation in the pediatric intensive care unit (PICU).

## 2. Case Report

A 1-year-old, 10 kilogram (kg), previously healthy boy presented to the pediatric emergency room after sustaining an accidental 24% total body surface 2nd degree scald burn from boiling water to his face, chest, arm, and thigh. He required emergent intubation during his initial debridement for increased work of breathing, stridor, and concern for thermal inhalation injury. His initial hospital course was complicated on hospital day (HD) 2 by fever of 39.4 degrees Celsius and subsequent pneumonia (*Moraxella catarrhalis, Streptococcus pneumoniae, and Haemophilus influenzae*) diagnosed by chest X-ray and bronchioalveolar lavage (BAL) culture obtained by bronchoscopy. He completed a ten-day treatment course of Meropenem for his pneumonia. Despite two lactate ringer boluses and adjustments to maintenance fluid rates to correct for dehydration with low urine output, he became hypotensive and required dopamine for 24 hours on HD 3. 

While intubated in the PICU, adequate sedation was especially important to prevent inadvertent extubation in the setting of an edematous airway. In addition, his endotracheal tube was sutured to his hard palate secondary to his facial burns which precluded securing it with tape. He required a combination of sedative and analgesic medications including fentanyl, morphine, and ketamine infusions as well as scheduled lorazepam. Inadequate sedation continued to complicate his course so methadone was initiated on HD 4. Despite the initiation of methadone, he required rescue doses of ketamine and midazolam, along with intermittent neuromuscular blockade with vecuronium from HD 2 to 10 to maintain safety. In preparation for removal of the endotracheal tube and in order to wean his medications with side effects of respiratory depression, a dexmedetomidine infusion was started. The infusion was initiated on HD 8 at 0.5 mcg/kg/hr and was increased briefly to a peak of 2.7 mcg/kg/hr on HD 10. In addition, clonidine was added on HD 10 which, in combination with the dexmedetomidine infusion, allowed discontinuation of the ketamine infusion and extubation that same day. Due to postextubation stridor, he received two short bursts of dexamethasone on HD 10 to 12 and HD 14 to 15. The dexmedetomidine infusion was slowly weaned off on HD 15 after 6.5 days of continuous infusion, and he was transferred to a general pediatric floor on HD 16.

On HD 18, the patient's morphine patient-controlled analgesia (PCA) was discontinued, oral clonidine was converted to an equivalent patch, and lorazepam was spaced to every six hours dosing in preparation for discharge. The next morning (HD 19), he was lethargic on exam; so, his clonidine patch, methadone, and lorazepam were discontinued as potential causative agents. At that time, he was afebrile and his vital signs were stable with blood pressures 79/48 to 95/55 mmHg, heart rate 90 to 108 beats/min, and respiratory rate 22 to 24 breaths/min. His physical exam was significant for burns in various stages of healing. Despite discontinuation of all analgesic and sedative medications, he remained lethargic and developed hypotension to 59/38 mmHg and tachycardia to 112 beats/min which responded to a 20 cc/kg normal saline bolus. A sepsis workup was obtained including a complete blood count, basic metabolic panel, C-reactive protein (CRP), urinalysis, and cultures of blood and urine. Labs were unremarkable with normal white blood cell count, CRP, and electrolytes and negative cultures. 

The possibility of adrenal insufficiency as a cause of hypotension was considered. The random cortisol level obtained at 4:49 am on HD 20 was low at 0.4 mcg/dL. Repeat labs were sent on HD 21, and his baseline cortisol at 9:47 am was 0.4 mcg/dL, and the cortisol level 60 minutes post-ACTH (125 mcg) stimulation test was also low at 7.8 mcg/dL (a level less than 18 mcg/dL is diagnostic of adrenal insufficiency). A repeat cortisol level at 8:45 am on HD 22 was again low at 1.5 mcg/dL. He was discharged to home on HD 23 on 8.5 mg/m^2^/day of hydrocortisone. He had a repeat cortisol level and ACTH stimulation test two months following hospital discharge which showed resolution of his adrenal insufficiency with a baseline cortisol level of 7.6 mcg/dL at 10:55 am and an appropriate 60 minute post-ACTH cortisol level of 28 mcg/dL.

## 3. Discussion

Adrenal insufficiency in the pediatric intensive care unit can be secondary to preexisting adrenal insufficiency, suppression of cortisol and ACTH production during critical illness, and drug-mediated changes to adrenal steroidogenesis [12]. Our patient's low cortisol level and inadequate response to ACTH stimulation are diagnostic of adrenal insufficiency. The temporary nature of our patient's adrenal insufficiency rules out a preexisting adrenal insufficiency. Although it is possible that his adrenal insufficiency was caused by his critical illness, most studies describing this entity occur in sepsis which this patient did not experience [12, 13]. In addition, the timing of the adrenal insufficiency in this patient (HD 19–23) was long after the critical stages of his illness, making this unlikely to be the cause of his adrenal insufficiency. 

We believe drug-induced adrenal insufficiency is most likely responsible for this patient's transient adrenal insufficiency. He received three medications that have been associated with adrenal insufficiency including dexmedetomidine, dexamethasone, and clonidine. In our patient, dexamethasone likely did not cause primary adrenal insufficiency because he received supraphysiologic dosing for a duration too brief to cause the adrenal atrophy responsible for the inappropriate response to an ACTH stimulation test that he exhibited [12]. Clonidine has been shown to decrease secretion of cortisol and ACTH in normal adults; so, we cannot exclude the possible role of clonidine in our patient's adrenal insufficiency [14]. However, there have not been extensive reports of clinical adrenal insufficiency with clonidine despite its extensive use at doses at or above that used in this patient. Therefore, even if clonidine played a role in the adrenal insufficiency in this patient, it seems likely that the treatment with dexmedetomidine was also involved, given this patient's symptomatic presentation with hypotension. 

The time course of our patient's symptomatic adrenal insufficiency (4 days following the discontinuation of the dexmedetomidine infusion) also supports the hypothesis that his adrenal insufficiency was due to the combined effects of clonidine and dexmedetomidine. Dexmedetomidine has a half-life of 2 hours and should be completely eliminated from the body in 12 hours, whereas a clonidine patch has a half-life of 21 hours [3, 15, 16]. The combination of clonidine and dexmedetomidine has become increasingly common, but clinically significant adrenal insufficiency has not been described before, most likely because the pharmacokinetics and dosing are different depending on age. A study by Vilo et al. investigating the pharmacokinetics of dexmedetomidine in children found that the half-life was longer in children younger than 2 years, like our patient [17]. Patients younger than 2 years also required larger initial doses of dexmedetomidine than older children due to their larger volume of distribution [17]. Our patient required high doses (peak of 2.7 mcg/kg/hr) for a long duration (6.5 days) which has been reported in adult and pediatric populations safely but dexmedetomidine is only FDA approved in adults for a continuous infusion of 0.7 mcg/kg/hr for 24 hours and is not yet approved in patients less than 18 years old [1, 4, 10, 18–20].

In summary, we report the possible association of continuous dexmedetomidine infusions and the development of adrenal insufficiency in the PICU. More research is needed to examine the development of adrenal insufficiency in the pediatric population, especially at higher and longer durations than the ones that have been FDA approved in pediatric or adult patients. 

## Abbreviations

mcg: Micrograms
kg: Kilogram
hr: Hour
dL: Deciliter
FDA: Food and Drug Administration
GABA: *γ*-Amino butyric acid
ACTH: Adrenocorticotrophic hormone
PICU: Pediatric Intensive Care Unit
HD: Hospital day
BAL: Bronchioalveolar lavage
mmHg: Millimeters of mercury
PCA: Patient-controlled analgesia
CRP: C-reactive protein
cc: Cubic centimeters.
